# Exploring the utility of N-acetylcysteine for loss of control eating: protocol of an open-label single-arm pilot study

**DOI:** 10.1186/s40814-025-01598-5

**Published:** 2025-02-19

**Authors:** Muthmainah Muthmainah, Diana Sketriene, Roberta G. Anversa, Emily Harris, Scott Griffiths, Andrea Gogos, Priya Sumithran, Robyn M. Brown

**Affiliations:** 1 The Florey Institute of Neuroscience and Mental Health, Victoria, Australia; 2 The Florey Department of Neuroscience and Mental Health, University of Melbourne, Victoria, Australia; 3 Department of Anatomy, Faculty of Medicine, Universitas Sebelas Maret, Surakarta, Indonesia; 4 Department of Biochemistry and Pharmacology, University of Melbourne, Level 8, Medical Building 181, Victoria, 3010 Australia; 5 Melbourne School of Psychological Sciences, University of Melbourne, Victoria, Australia; 6 School of Translational Medicine, Monash University, Victoria, Australia; 7 Department of Endocrinology and Diabetes, Alfred Health, Victoria, Australia

**Keywords:** Compulsive eating, *N*-acetylcysteine, Ecological momentary assessment, Stress, Mood, Food addiction, Addictive eating, Binge eating, Emotional eating, Stress eating

## Abstract

**Background:**

A sense of loss of control over eating, such that eating occurs despite the intent not to, is common in people with obesity and eating disorders such as binge eating disorder and bulimia nervosa. Currently, options for management of loss of control eating are limited. We recently determined that the pro-drug N-acetylcysteine (NAC) reduces compulsive-like eating in a rat model of diet-induced obesity. We will now conduct a single site, open-label pilot study to examine the feasibility of a randomized controlled trial (RCT) of NAC for loss of control eating in humans.

**Methods:**

Thirty-six adult volunteers with loss of control eating will be enrolled. All participants will receive NAC at a dose of 1200 mg orally twice daily for 12 weeks. Eating behaviors and triggers will be assessed before and after the NAC treatment period using questionnaires (Eating Loss of Control Scale, Palatable Eating Motives Scale: Coping Subscale, Food Craving Inventory, Reward-Based Eating Scale, Perceived Stress Scale, and Emotional Eating Scale) and ecological momentary assessment (EMA). The primary outcomes of this feasibility study are recruitment rate, participant retention rate at week 12, and medication adherence. The secondary outcome is change in Eating Loss of Control Scale score from baseline to week 12. Exploratory data will be collected on the change in eating behaviors from baseline to week 12. Although EMA can provide real-time data on eating behaviors compared with retrospective questionnaires, it relies on repeated daily measurement for long periods which can affect participant’s adherence to study protocol. Therefore, this feasibility study will assess the performance of EMA versus retrospective questionnaires and will determine which approach suits the purposes of the research.

**Discussion:**

The results of this study will inform the feasibility of a RCT of NAC for loss of control eating using EMA.

**Trial registration:**

This study was prospectively registered with the Australian and New Zealand Clinical Trials Registry in June 2022 (ACTRN12622000902796).

## Introduction

### Background

Eating behavior is controlled by both homeostatic and non-homeostatic processes. For some people, however, eating behavior is not always driven by homeostatic need and can become compulsive in nature [1]. People with compulsive eating experience a sense of loss of control over their eating behavior, i.e., eating despite the intent or desire not to and find it difficult to stop eating [1, 2]. This eating is often habitual (e.g., mindless eating) and occurs in response to emotional triggers (e.g., when feeling angry, down, bored, or stressed), food-associated cues (e.g., the smell or sight of food), and contexts (e.g., celebratory events). They also tend to think about food a lot (preoccupation, colloquially known as “food noise”), and they may frequently experience cravings for certain foods [1–3]. Though not a clinical diagnosis, loss of control eating can have a negative impact on health and on the outcomes of behavioral, medical, and surgical obesity treatment [4–7]. Loss of control eating is found in both children and adult with 42–55% of children aged 8–13 years old and 21–33% of men and women reporting episodes of loss of control eating [1].

Compulsive eating is a common feature of obesity and eating disorders (such as binge eating disorder and bulimia nervosa [8]); hence, treatment for compulsive eating may potentially offer shared therapeutic benefits for these conditions. However, treatment options targeting this behavior are limited. Psychological intervention such as cognitive behavioral therapy (CBT) is considered the first line of treatment [9]. Currently, there is only one medication approved for the treatment of any form of compulsive eating and that is lisdexamfetamine, a stimulant which is FDA-approved for binge eating disorder. However, being an amphetamine medication, lisdexamfetamine comes with a risk of abuse [10]. Further, cardiovascular side effects including palpitations and increased blood pressure have been reported, as well as sleep disturbances. These undesirable effects contribute to medication discontinuation and can represent a challenge for the wider use of this drug [9]. Stimulant medications may improve compulsive eating through their dual effects on reward and executive function. Stimulants increase the availability of catecholamine to improve executive function. They also act on the reward system to suppress appetite which is associated with increased extracellular level of dopamine in the nucleus accumbens [11]. Several medications, such as antidepressants (e.g., fluoxetine), anticonvulsants (e.g., topiramate), and opioid antagonists (e.g., naltrexone) have been proposed as potential candidates for the treatment of compulsive eating [10, 11], though further research is needed to establish their efficacy for this purpose. Many pharmacotherapies used in clinical trials for compulsive eating have not shown high efficacy in reducing symptomatology (e.g., the frequency of binge eating), and the wider use of some of these drugs is limited by their adverse effects [11]. Findings from RCT testing antidepressant drugs including fluvoxamine, duloxetine, buproprion, and desipramine either given as monotherapy or in combination with other intervention (e.g., CBT) have failed to significantly improve binge eating outcome [12]. The anticonvulsant, topiramate, has been shown to reduce binge eating compared to placebo [12–14] and enhance CBT outcomes for binge eating [15]. However, the wider use of topiramate is limited by its high rates of adverse effects and dropout rates (26–68% dropout) [11] suggesting that further investigation of potential pharmacotherapies for compulsive eating is warranted. Based on neuropharmacological systems involved in compulsive eating, several pharmacotherapy options have been investigated including medications that target the cholinergic system, orexin system, glutamatergic system, trace amine-associated receptor-1, cannabinoid receptor 1, and the sigma-1 receptor [2].

There are several lines of evidence to suggest that N-acetylcysteine (NAC) could be of potential benefit for compulsive eating. NAC, a cysteine prodrug, has been shown to reduce several compulsive behaviours, such as use of addictive drugs and compulsive hair pulling and skin picking [16]. Abnormal regulation of glutamate in the striatum has been implicated in the pathophysiology underlying compulsive behaviors [17, 18]. Therefore, the mechanism of action of NAC may involve modulation of the cysteine-glutamate antiporter, known as system xc-, and the glial glutamate transporter, GLT-1, which play a key role in glutamate homeostasis and influence glutamate transmission [19]. Repeated use of drugs of abuse such as cocaine leads to persistent reduction of cysteine-glutamate exchange via system xc- in brain areas involved in hedonic motivated behaviour such as the nucleus accumbens [20–23] which disrupts the level of extracellular glutamate. Similarly, drugs of abuse also reduce the expression of GLT-1 in the nucleus accumbens [20, 21]. Administration of NAC restores xc-function and ameliorates drug-seeking behavior [23–25]. Likewise, NAC administration upregulates GLT-1 expression [21, 26] which mediates NAC inhibition of cue-induced reinstatement of cocaine seeking [26]. Restoring extracellular glutamate with NAC has been shown to inhibit excitatory synaptic activity in the nucleus accumbens via activation of group II metabotropic glutamate receptors (mGluR2/3) [27]. In humans, NAC treatment at 2400 mg/day for 4 weeks reduces tobacco use (32 participants in total) [20] and cocaine use (23 participants in total) [28]. Our laboratory has shown that NAC reduces compulsive eating behaviour in diet-induced obese rats [29]. NAC has also been shown to reduce binge-like eating in a model where rats were given daily limited access (30 min) to a highly palatable diet [30] and reduce food-seeking behavior in a rat model of cue-induced reinstatement [31]. As yet, there are no studies examining the effect of NAC on compulsive eating in humans. One small study has examined the effects of NAC on eating behavior in people with bulimia nervosa [32]. This open-label study examined the effect of NAC on binge-purge frequency in eight participants, of whom only two completed the 12-week intervention (the following reasons for attrition were reported: loss to follow up, lack of efficacy, or experiencing side effects [32], thus limiting the conclusions that can be drawn. Therefore, more research is required to evaluate the efficacy of NAC for compulsive eating.

Here, we are conducting a pilot study to explore the utility of NAC for the treatment of loss of control eating. We will employ ecological momentary assessment (EMA) to monitor treatment response. EMA can capture valuable real-time information on eating behaviors compared with retrospective questionnaires but requires more resources and time commitment for data collection. Therefore, this feasibility study will assess whether this approach suits the purposes of the research.

### Aims

The primary aim of this study is to assess the feasibility of delivering a 12-week NAC intervention in people with loss of control eating. The secondary objective is to estimate the clinical efficacy of NAC to inform a sample size calculation for future research. In addition, we aim to explore the performance of self-reported measures of eating behaviors and mood using EMA compared with retrospective questionnaires.

## Methods/design

### Design

**Fig. 1:**
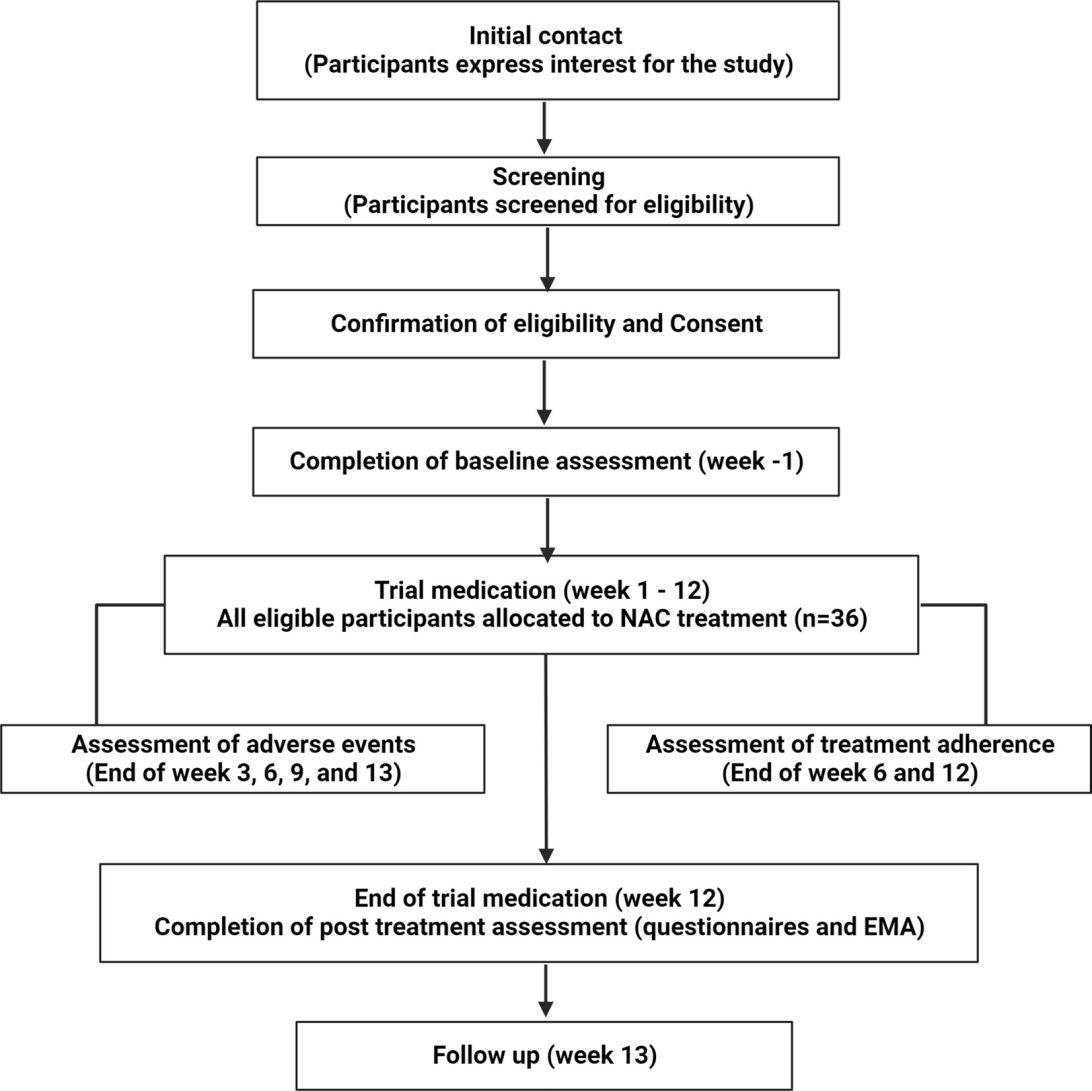
Trial flow diagram

This is an open-label single-arm pilot study to examine the feasibility of recruiting and retaining participants during a 12-week NAC intervention in people with loss of control eating. After completing the baseline assessments, all participants will receive NAC orally at a daily dose of 2400 mg (two capsules of 600 mg, twice daily) for 12 weeks. A follow up assessment will be conducted after treatment using the same measures as in the baseline assessment. The trial flow diagram (Fig. 1) depicts the overall procedure in the study. This single-site study will be conducted at the Florey Institute of Neuroscience and Mental Health, Melbourne, Victoria, Australia.

### Study population

Community residents in greater Melbourne will be recruited to participate. Inclusion criteria are all of the following: (1) age ≥18 years (only adults will be included in the study because children required different measures for outcomes assessment) and (2) loss of control eating (defined as at least one episode of loss of control eating in the past month as assessed using the Eating Loss of Control Scale) [33]. Our definition for loss of control eating is based on previous studies which have used a similar cut-off point for their inclusion criteria [34–37]. The exclusion criteria include (1) cognitive impairment which will hinder ability to provide informed consent or to complete the questionnaires, (2) pregnant or lactating or planning to become pregnant during the study, (3) use of any medication containing NAC or other contraindicated medications within 4 weeks prior to enrolment (such as carbamazepine, nitroglycerin, anticoagulants, and antioxidant agents or glutathione prodrugs that can enhance the effect of NAC, e.g., selenium or vitamin E), (4) previous hypersensitivity to NAC, (5) known or suspected active systemic medical disorder or any serious medical illness that NAC may adversely affect (e.g., epilepsy, recent gastrointestinal bleeding, esophageal varices, renal stones, hepatic or renal failure, and asthma), (6) surgery within the past 28 days, and (7) currently using or will be using a formal weight loss program during the study.

### Sample size estimation

Sample size calculation was based on progression criterion for a pilot study as recommended by Consolidated Standards of Reporting Trials CONSORT [38]. Retention rate, defined as the percentage of individuals who complete the retrospective questionnaires at week 12, was used for sample size calculation. A total sample of 36 participants are required assuming at least 80% of enrolled participants are retained in the study compared to a worst-case retention rate of 60% or less in a one sample *t*-test with 80% power at *p* <0.05 based on the exact binomial test. The cut-off points of 60% and 80% set as the “stop” and “go” criteria provides sufficient sample size and power for the assessment of the primary feasibility outcome with the lowest expected denominator (e.g., retention rate).

### Intervention

NAC will be administered orally at a daily dose of 2400 mg in capsule formulation for 12 weeks. It will be taken as two capsules of 600 mg twice daily, in the morning and evening with food. A systematic review of clinical trials of NAC found that at a dose of 2400 mg/day, NAC is well tolerated and effective for treating a range of compulsive and addictive behaviors including obsessive compulsive disorder and drug use and craving (e.g., for methamphetamine and cannabis) [16]. A daily dose of up to 3600 mg/day has been shown to be safe and tolerable [16]. The 10-item Medication Adherence Rating Scale [39], as well as pill count at the end of weeks 6 and 12, will be used to determine adherence with the study medication. Weekly interactions via phone calls or text messages between participants and the investigators will be conducted to maintain adherence to medication and study protocol.

### Recruitment and screening

Participants will be recruited via mailing lists and social media advertisements. Participants will express interest via an electronic form (REDCap) or by contacting the study researcher via email and will then be provided with the plain language statement and consent form. Prospective participants will complete an online questionnaire to screen eligibility. Participants who meet the requirements based on this initial screening will be invited to the study site at the Florey Institute of Neuroscience and Mental Health, Melbourne, Victoria, for further assessment to confirm eligibility.

### Assessment schedules

Prior to performing any study-specific procedure, written consent will be obtained for each participant. A study researcher will provide participants with the plain language statement and consent form. This document will describe the purpose of the study, the procedures to be followed, and the risks and benefits of participation. The study researcher will check that the participant comprehends the information provided and will answer any questions the participant has about the study. Participants will be given time to consider their involvement and an opportunity to discuss with relevant others. Participants will then be invited to provide written consent. Consent will be voluntary and free from coercion. When the eligibility of the participant has been confirmed, the participant will be assigned to intervention. The schedule of assessments is described in Table 1.

**Table 1:** Schedule of assessments

TIME POINT	STUDY PERIOD				
	**Enrolment**	**Intervention period**			**Post-intervention**
	***Week-***_***1***_	***Week***_***1–4***_	***Week***_***5–8***_	***Week***_***9–12***_	***Week***_***13***_
**ENROLMENT**					
Eligibility screen	X				
Informed consent	X				
**INTERVENTIONS**					
NAC Intervention		X	X	X	
**ASSESSMENTS**					
Demographic characteristics	X				
Questionnaires*	X			X	
EMA survey#	X			X	
Monitoring of adverse effects		X	X	X	X
Medication adherence			X	X	

*Eating Loss of Control Scale, Palatable Eating Motives Scale, Food Craving Inventory, Reward-Based Eating Scale, Perceived Stress Scale, Emotional Eating Scale

^#^Last for a period of 7 days

### Safety monitoring

NAC has a good safety profile. Safety and monitoring will be overseen by the investigators (including medically trained personnel) who will regularly review adverse events. An independent medical monitor has been appointed to provide further safety oversight.

Adverse events and adverse reactions (non-serious and serious) will be captured from the time of administration of NAC until 7 days after the final dose and will be followed until resolution or stabilization. Assessment of any medically related changes in the participant’s well-being will be made at the end of weeks 3, 9, and 13 using open-ended questions via an online survey. In addition, in-person assessment will be done at the end of week 6 during the second visit to the study site. Each adverse event will be counted once for a given participant. If the same event is reported more than once, the event counted will be the event with the highest severity (coded mild, moderate, or severe). In the event that serious adverse effects occur, NAC medication will be suspended, and adequate medical care will be provided to the participant.

### Data management

Hard copy data will be stored in a locked cabinet in a secure workspace at the University of Melbourne, accessible to the research team only. Electronic data will be securely stored in the Florey Institute’s REDCap database system and secure network file servers, which are backed up regularly. The permissions granted to each user within the REDCap project will be controlled by the investigators. For EMA, data will be stored at the secure SEMA^3^ server housed at the University of Melbourne. Each participant is assigned a unique participant ID at enrolment. Data containing private or confidential information or data that can identify a participant will be accessible only by designated members of the research team. Following the completion of the study and data analysis, the data will be retained for the mandatory archive period (15 years).

### Outcome measures

The primary outcomes are participant retention rate, recruitment rate, and medication adherence. Retention rate will be calculated as the proportion of individuals who completed retrospective questionnaires at week 12. Recruitment rate will be assessed by calculating the number of recruited participants per month. Adherence to medication will be the percentage of doses taken, based on capsule count. The secondary outcome is change in Eating Loss of Control Scale score from baseline to week 12. The exploratory outcomes include the following:

- Adherence with EMA protocol (percentage of EMA surveys completed; percentage of participants who completed at least 80% of EMA surveys);
- Concordance between EMA and retrospective measures (internal consistency, convergent validity, and sensitivity to detect treatment outcome);
- Change of scores in the drivers of eating and eating behaviour from baseline to week 12:
- Emotional eating (Palatable Eating Motives Scale: Coping subscale and Emotional Eating Scale);
- Food craving (Food Craving Inventory);
- Preoccupation with food (Reward-based Eating Scale);
- Perceived stress (Perceived Stress Scale).

The outcomes will be assessed using both retrospective questionnaires (described below) and EMA.

#### Questionnaires

Sixvalidated questionnaires, including the Eating Loss of Control Scale [33], Palatable Eating Motives Scale: Coping Subscale [40], Food Craving Inventory [41], Reward-Based Eating Scale [42], Perceived Stress Scale [43], and Emotional Eating Scale [44], will be administered during baseline and post-NAC treatment assessment. Participants will be asked to complete these questionnaires electronically.

#### EMA measures

EMA survey will be delivered via an application installed on the participants’ mobile phone (SEMA^3^, University of Melbourne). Survey prompts will be sent three times per day (at semi-random times) over 7 days before and after NAC treatment. EMA questions will be modified from validated questionnaires including items from the Perceived Stress Scale (for stress level), Emotional Eating Scale (for mood/emotion), and Food Craving Inventory (for craving experience). Questions for loss of control eating were adapted from a previous study [45]. Mood/emotion, craving, perceived stress, loss of control eating, and preoccupation with food will be rated on a Likert scale consisting of a horizontal rating slider (1=*not at all* to 5=*extremely*), and exposure to specific food cues will require a yes/no answer.

### Handling of missing data

Participants with missing data on post-intervention outcome assessment (retrospective questionnaire) will be excluded from analysis.

### Statistical analysis

All participants who received at least the first dose of study drug and provided at least 1 post baseline assessment will be included in the analysis. Data will be cleaned and checked for accuracy. Demographic characteristics will be summarized as proportions for categorical data and means and standard deviations for continuous data or medians as appropriate. For primary outcomes, retention rate will be calculated as the proportion of individuals who completed retrospective questionnaires at week 12; recruitment rate will be calculated as the total number of participants recruited divided by the total number of months that the trial recruited for. Secondary and exploratory outcomes will be reported as estimates of effects (95% confidence interval). Pearson or Spearman correlation will be used to analyze the concordance between EMA and retrospective measures (convergent validity), and reliability (internal consistency) will be examined with Cronbach’s alpha. Compliance rate will be estimated from the average number of EMA surveys completed out of all administered surveys.

Adverse events will be reported as the number and percentage of participants reporting adverse events. Other information about each adverse event including start date, stop date, severity, relationship, expectedness, outcome, and duration, including adverse events leading to premature discontinuation from the trial intervention and serious treatment-emergent adverse events, will also be collected.

## Discussion

This study will test the feasibility of delivering a 12-week NAC intervention and assessing responses using EMA for 7 days before and after the intervention in people with loss of control eating. Treatment outcomes related to eating behaviour are commonly evaluated using retrospective questionnaires. The disadvantage of this approach is the potential influence of recall bias. EMA is typically administered via a smartphone-based application with multiple sampling (i.e., several times per day over days to weeks) and provides close to real-time data. This may be advantageous in detecting associations between factors involved in eating behavior, especially those with a dynamic nature such as stress and mood. In the field of eating disorders, there has been a substantial growth in the number of studies employing EMA in the last few years. These studies have focused on understanding the theoretical model of eating disorders, examining the antecedents/predictors and consequences of eating behaviors, and understanding the relationship between variables implicated in eating disorder [46]. Notably, the majority of studies using EMA are observational and have not employed this assessment in the context of detecting treatment outcomes. EMA has been used to study treatment outcomes in some psychiatric disorders such as substance use disorder, anxiety disorder, depression, attention-deficit hyperactivity disorder (ADHD), and psychotic disorders [47]. During antidepressant treatment in patients with major depressive disorder, EMA was able to detect subtle early changes in affect that were predictive of later response and remission [48]. Another advantage of using EMA during treatment is its sensitivity to detect context-specific treatment effects. An EMA study in smokers with ADHD showed that ADHD medication improved concentration specifically during stress and smoking abstinence [49]. Moreover, EMA is more sensitive than retrospective questionnaires in capturing changes in health-related quality of life following cardiac interventions [50]. This study will be among the first to employ EMA to measure the effects of an intervention on loss of control eating. However, the reliance of EMA on repeated daily measurement for long periods may be burdensome for participants and affect adherence rate. Thus, comparing the result of EMA measures with retrospective questionnaires will allow researchers to choose between the approaches that best fit with the study objectives when conducting future studies. Studies on feasibility of EMA commonly look at participants’ acceptability of the assessment, response rate to survey prompts, retention rate, initial validity and reliability of EMA data, and identification of technical issues which may arise during the study [51–53]. Our pilot study will collect this information as a basis for designing a future RCT.

This study will investigate the use of NAC for the treatment of loss of control eating in adults by examining the response to a dose of 2400 mg/day for 12 weeks. A recent systematic review on clinical trials of NAC found that at 2400 mg/day [16], NAC was well tolerated and effective for treating a range of compulsive and addictive behaviors including obsessive compulsive disorder and drug use and craving (e.g., for methamphetamine and cannabis). A daily dose of up to 3600 mg/day of NAC has been shown to be safe and tolerable [16, 54]. Data collected from the trial will help improve our understanding of the potential utility of NAC to reduce compulsive eating. If it is found to be feasible, further larger scale studies to determine efficacy of the intervention will be undertaken.

Compulsive eating is a hallmark of some eating disorders including binge eating disorder [55, 56]. Approximately 3–5% of individuals with obesity in the general population and up to 30% of people seeking treatment for obesity have compulsive eating [57–59]. Compulsive eating is also the core feature of so-called “food addiction” that affects 5–15% of people in the community as measured by the Yale Food Addiction Scale (YFAS) [60–62]. Therefore, if proven efficacious, NAC may serve as an inexpensive, safe adjunct in the management of compulsive eating that could potentially benefit people with a broad range of conditions.

This study has some limitations. Firstly, we do not exclude people who use drugs that may affect appetite and eating behaviors, such as topiramate, as the primary goal of this pilot study is to determine the feasibility of delivering a 12-week intervention of NAC with a 7-day EMA assessment delivered 3x daily during this intervention. Medications with demonstrated effects on compulsive eating behaviours (such as lisdexamfetamine) will be excluded from a subsequent phase 3 trial examining the efficacy of NAC. Secondly, our definition of loss of control eating is more inclusive and does not satisfy the criteria for an eating disorder diagnosis. However, a low level of inclusion criterion (i.e., 1–2 episodes per month) is used by other studies when trialling possible interventions for compulsive eating [34–37]. Thus, our definition is consistent with other studies. Thirdly, we conducted an open label study with no control arm. Uncontrolled trials can produce greater estimates of the mean treatment effect; thus, they may inflate the expectations from the intervention. Due to this potential bias, efficacy results from uncontrolled trials are not as valid as RCTs and have limited generalizability [63, 64]. However, in this pilot study, our focus is not evaluating the efficacy of N-acetylcysteine. Instead, we are evaluating the feasibility of delivering the intervention and adherence to the intervention and assessments. This information is critical for designing a future RCT to empirically determine the efficacy of N-acetylcysteine for the treatment of compulsive eating. Hence, a single-arm design is appropriate for this purpose. This design is pragmatic and allows for smaller sample size. Fourthly, the use of self-reported data for assessment of certain outcomes may introduce potential measurement bias (e.g., for eating behavior).

## Conclusion

There is currently a lack of effective treatments available for compulsive eating. The results of this open-label pilot study will inform (1) the feasibility of a delivering a 12-week intervention of NAC in people with loss of control eating, (2) the feasibility of a 3x per day/7-day EMA assessment during NAC intervention, and (3) the potential utility of NAC for loss of control eating.

## Data Availability

Not applicable.

## References

[1] Anversa RG, et al. A review of sex differences in the mechanisms and drivers of overeating. Front Neuroendocrinol. 2021;63: 100941. 34454955 10.1016/j.yfrne.2021.100941

[2] Moore CF, et al. Neuropharmacology of compulsive eating. Philos Trans R Soc Lond B Biol Sci. 2018;373(1742):20170024. 29352024 10.1098/rstb.2017.0024 PMC5790823

[3] Chao AM, et al. Food craving, binge eating, and eating disorder psychopathology: exploring the moderating roles of gender and race. Eat Behav. 2016;21:41–7. 26741258 10.1016/j.eatbeh.2015.12.007 PMC4851566

[4] White MA, et al. Loss of control over eating predicts outcomes in bariatric surgery patients: a prospective, 24-month follow-up study. J Clin Psychiatry. 2010;71(2):175–84. 19852902 10.4088/JCP.08m04328blu PMC2831110

[5] Goldschmidt AB. Are loss of control while eating and overeating valid constructs? A critical review of the literature. Obes Rev. 2017;18(4):412–49. 28165655 10.1111/obr.12491 PMC5502406

[6] Manasse SM, et al. Are individuals with loss-of-control eating more prone to dietary lapse in behavioural weight loss treatment? An ecological momentary assessment study. Eur Eat Disord Rev. 2018;26(3):259–64. 29484774 10.1002/erv.2583 PMC5916047

[7] Potenza MN, Grilo CM. How relevant is food craving to obesity and its treatment? Front Psychiatry. 2014;5:164. 25477827 10.3389/fpsyt.2014.00164 PMC4237037

[8] Moore CF, et al. Neuroscience of compulsive eating behavior. Front Neurosci. 2017;11:469. 28883784 10.3389/fnins.2017.00469 PMC5573809

[9] Appolinario JC, Nardi AE, McElroy SL. Investigational drugs for the treatment of binge eating disorder (BED): an update. Expert Opin Investig Drugs. 2019;28(12):1081–94. 31714807 10.1080/13543784.2019.1692813

[10] Levitan MN, et al. Binge eating disorder: a 5-year retrospective study on experimental drugs. J Exp Pharmacol. 2021;13:33–47. 33542663 10.2147/JEP.S255376 PMC7853418

[11] Boswell RG, Potenza MN, Grilo CM. The neurobiology of binge-eating disorder compared with obesity: implications for differential therapeutics. Clin Ther. 2021;43(1):50–69. 33257092 10.1016/j.clinthera.2020.10.014 PMC7902428

[12] Reas DL, Grilo CM. Pharmacological treatment of binge eating disorder: update review and synthesis. Expert Opin Pharmacother. 2015;16(10):1463–78. 26044518 10.1517/14656566.2015.1053465 PMC4491373

[13] McElroy SL, et al. Topiramate for the treatment of binge eating disorder associated with obesity: a placebo-controlled study. Biol Psychiatry. 2007;61(9):1039–48. 17258690 10.1016/j.biopsych.2006.08.008

[14] Nourredine M., et al., Efficacy and safety of topiramate in binge eating disorder: a systematic review and meta-analysis. CNS Spectr. 2021;26(5):459–67. 10.1017/S1092852920001613 32641176

[15] Claudino AM, et al. Double-blind, randomized, placebo-controlled trial of topiramate plus cognitive-behavior therapy in binge-eating disorder. J Clin Psychiatry. 2007;68(9):1324–32. 17915969 10.4088/jcp.v68n0901

[16] Deepmala, et al., Clinical trials of N-acetylcysteine in psychiatry and neurology: a systematic review. Neurosci Biobehav Rev. 2015;55:294–321. 10.1016/j.neubiorev.2015.04.015 25957927

[17] Kariuki-Nyuthe C, Gomez-Mancilla B, Stein DJ. Obsessive compulsive disorder and the glutamatergic system. Curr Opin Psychiatry. 2014;27(1):32–7. 24270485 10.1097/YCO.0000000000000017

[18] Brown RM, Kupchik YM, Kalivas PW. The story of glutamate in drug addiction and of N-acetylcysteine as a potential pharmacotherapy. JAMA Psychiat. 2013;70(9):895–7. 10.1001/jamapsychiatry.2013.2207 23903770

[19] Bridges R, et al. Thinking outside the cleft to understand synaptic activity: contribution of the cystine-glutamate antiporter (System xc-) to normal and pathological glutamatergic signaling. Pharmacol Rev. 2012;64(3):780–802. 22759795 10.1124/pr.110.003889 PMC3400835

[20] Knackstedt LA, et al. The role of cystine-glutamate exchange in nicotine dependence in rats and humans. Biol Psychiatry. 2009;65(10):841–5. 19103434 10.1016/j.biopsych.2008.10.040 PMC2756612

[21] Knackstedt LA, Melendez RI, Kalivas PW. Ceftriaxone restores glutamate homeostasis and prevents relapse to cocaine seeking. Biol Psychiatry. 2010;67(1):81–4. 19717140 10.1016/j.biopsych.2009.07.018 PMC2795043

[22] Kau KS, et al. Blunted cystine-glutamate antiporter function in the nucleus accumbens promotes cocaine-induced drug seeking. Neuroscience. 2008;155(2):530–7. 18601982 10.1016/j.neuroscience.2008.06.010 PMC2614296

[23] Baker DA, et al. Neuroadaptations in cystine-glutamate exchange underlie cocaine relapse. Nat Neurosci. 2003;6(7):743–9. 12778052 10.1038/nn1069

[24] Madayag A, et al. Repeated N-acetylcysteine administration alters plasticity-dependent effects of cocaine. J Neurosci. 2007;27(51):13968–76. 18094234 10.1523/JNEUROSCI.2808-07.2007 PMC2996827

[25] Amen SL, et al. Repeated N-acetyl cysteine reduces cocaine seeking in rodents and craving in cocaine-dependent humans. Neuropsychopharmacology. 2011;36(4):871–8. 21160464 10.1038/npp.2010.226 PMC3052624

[26] Reissner KJ, et al. Glutamate transporter GLT-1 mediates N-acetylcysteine inhibition of cocaine reinstatement. Addict Biol. 2015;20(2):316–23. 24612076 10.1111/adb.12127 PMC4437505

[27] Moran MM, et al. Cystine/glutamate exchange regulates metabotropic glutamate receptor presynaptic inhibition of excitatory transmission and vulnerability to cocaine seeking. J Neurosci. 2005;25(27):6389–93. 16000629 10.1523/JNEUROSCI.1007-05.2005 PMC1413952

[28] Mardikian PN, et al. An open-label trial of N-acetylcysteine for the treatment of cocaine dependence: a pilot study. Prog Neuropsychopharmacol Biol Psychiatry. 2007;31(2):389–94. 17113207 10.1016/j.pnpbp.2006.10.001

[29] Sketriene D, et al. N-acetylcysteine reduces addiction-like behaviour towards high-fat high-sugar food in diet-induced obese rats. Eur J Neurosci. 2021;54(3):4877–87. 34028895 10.1111/ejn.15321

[30] Hurley MM, et al. N-acetylcysteine decreases binge eating in a rodent model. Int J Obes (Lond). 2016;40(7):1183–6. 26975440 10.1038/ijo.2016.31 PMC4935583

[31] Ramirez-Niño AM, D’Souza MS, Markou A. N-acetylcysteine decreased nicotine self-administration and cue-induced reinstatement of nicotine seeking in rats: comparison with the effects of N-acetylcysteine on food responding and food seeking. Psychopharmacology. 2013;225(2):473–82. 22903390 10.1007/s00213-012-2837-3 PMC3697766

[32] Guerdjikova AI, et al. N-acetylcysteine in bulimia nervosa–open-label trial. Eat Behav. 2013;14(1):87–9. 23265409 10.1016/j.eatbeh.2012.11.001

[33] Blomquist KK, et al. Development and validation of the eating loss of control scale. Psychol Assess. 2014;26(1):77–89. 24219700 10.1037/a0034729 PMC4021596

[34] Shomaker LB, et al. A randomized, comparative pilot trial of family-based interpersonal psychotherapy for reducing psychosocial symptoms, disordered-eating, and excess weight gain in at-risk preadolescents with loss-of-control-eating. Int J Eat Disord. 2017;50(9):1084–94. 28714097 10.1002/eat.22741 PMC5759342

[35] Goode RW, et al. The feasibility of a binge eating intervention in Black women with obesity. Eat Behav. 2018;29:83–90. 29549863 10.1016/j.eatbeh.2018.03.005 PMC5935580

[36] Keshen AR, et al. Effect of stimulant medication on loss of control eating in youth with attention deficit/hyperactivity disorder: a prospective, observational case series study protocol. J Eat Disord. 2022;10(1):152. 36320022 10.1186/s40337-022-00674-y PMC9628055

[37] ClinicalTrials.gov, Cognitive behavioural therapy for adolescent binge eating and loss of control eating. NCT04088097. Available from: https://classic.clinicaltrials.gov/ct2/show/NCT04088097

[38] Lewis M, et al. Determining sample size for progression criteria for pragmatic pilot RCTs: the hypothesis test strikes back: Pilot Feasibility Stud. 2021;7(1):40. 33536076 10.1186/s40814-021-00770-x PMC7856754

[39] Thompson K, Kulkarni J, Sergejew AA. Reliability and validity of a new Medication Adherence Rating Scale (MARS) for the psychoses. Schizophr Res. 2000;42(3):241–7. 10785582 10.1016/s0920-9964(99)00130-9

[40] Burgess EE, et al. Profiling motives behind hedonic eating. Preliminary validation of the Palatable Eating Motives Scale. Appetite. 2014;72:66–72. 24076018 10.1016/j.appet.2013.09.016

[41] White MA, et al. Development and validation of the food-craving inventory. Obes Res. 2002;10(2):107–14. 11836456 10.1038/oby.2002.17

[42] Mason AE, et al. Improving assessment of the spectrum of reward-related eating: the RED-13. Front Psychol. 2017;8:795. 28611698 10.3389/fpsyg.2017.00795 PMC5447741

[43] Cohen S, Kamarck T, Mermelstein R. A global measure of perceived stress. J Health Soc Behav. 1983;24(4):385–96. 6668417

[44] Arnow B, Kenardy J, Agras WS. The Emotional Eating Scale: the development of a measure to assess coping with negative affect by eating. Int J Eat Disord. 1995;18(1):79–90. 7670446 10.1002/1098-108x(199507)18:1<79::aid-eat2260180109>3.0.co;2-v

[45] Berg KC, et al. Negative affect prior to and following overeating-only, loss of control eating-only, and binge eating episodes in obese adults. Int J Eat Disord. 2015;48(6):641–53. 25808854 10.1002/eat.22401 PMC4543439

[46] Schaefer LM, Engel SG, Wonderlich SA. Ecological momentary assessment in eating disorders research: recent findings and promising new directions. Curr Opin Psychiatry. 2020;33(6):528–33. 32740204 10.1097/YCO.0000000000000639 PMC7780347

[47] Bos, F.M., R.A. Schoevers, and M. aan het Rot, Experience sampling and ecological momentary assessment studies in psychopharmacology: a systematic review. Eur Neuropsychopharmacol, 2015;25(11): 1853–64. 10.1016/j.euroneuro.2015.08.008 26336868

[48] Geschwind N, et al. Early improvement in positive rather than negative emotion predicts remission from depression after pharmacotherapy. Eur Neuropsychopharmacol. 2011;21(3):241–7. 21146375 10.1016/j.euroneuro.2010.11.004

[49] Gehricke JG, et al. ADHD medication reduces cotinine levels and withdrawal in smokers with ADHD. Pharmacol Biochem Behav. 2011;98(3):485–91. 21356232 10.1016/j.pbb.2011.02.021 PMC3065552

[50] Oreel TH, et al. Ecological momentary assessment versus retrospective assessment for measuring change in health-related quality of life following cardiac intervention. J Patient Rep Outcomes. 2020;4(1):98. 33196959 10.1186/s41687-020-00261-2 PMC7669938

[51] Jean, FA.M., et al., Feasibility and validity of Ecological Momentary Assessment in patients with acute coronary syndrome. BMC Cardiovasc Disord, 2020; 20(1): 499. 10.1186/s12872-020-01774-w. 10.1186/s12872-020-01774-w PMC7694267 33246420

[52] Moitra, E., et al., Feasibility and acceptability of post-hospitalization ecological momentary assessment in patients with psychotic-spectrum disorders. Compr Psychiatry, 2017;74:204–213. 10.1016/j.comppsych.2017.01.018 10.1016/j.comppsych.2017.01.018 PMC5369417 28231480

[53] Kirk GD, et al. The exposure assessment in current time study: implementation, feasibility, and acceptability of real-time data collection in a community cohort of illicit drug users. AIDS Res Treat, 2013; 2013: 594671. 10.1155/2013/594671. 10.1155/2013/594671 PMC3836292 24307943

[54] Schmaal L, et al. Efficacy of N-acetylcysteine in the treatment of nicotine dependence: a double-blind placebo-controlled pilot study. Eur Addict Res. 2011;17(4):211–6. 21606648 10.1159/000327682

[55] APA. Diagnostic and statistical manual of mental disorders. 5th ed. Washington DC: American Psychiatric Press; 2013.

[56] Kessler RM, et al. The neurobiological basis of binge-eating disorder. Neurosci Biobehav Rev. 2016;63:223–38. 26850211 10.1016/j.neubiorev.2016.01.013

[57] de França GV, Gigante DP, Olinto MT. Binge eating in adults: prevalence and association with obesity, poor self-rated health status and body dissatisfaction. Public Health Nutr. 2014;17(4):932–8. 23472839 10.1017/S1368980013000591 PMC10282444

[58] Ivezaj V, White MA, Grilo CM. Examining binge-eating disorder and food addiction in adults with overweight and obesity. Obesity (Silver Spring). 2016;24(10):2064–9. 27558207 10.1002/oby.21607 PMC5039112

[59] Kessler RC, et al. The prevalence and correlates of binge eating disorder in the World Health Organization World Mental Health Surveys. Biol Psychiatry. 2013;73(9):904–14. 23290497 10.1016/j.biopsych.2012.11.020 PMC3628997

[60] Davis C, et al. Evidence that “food addiction” is a valid phenotype of obesity. Appetite. 2011;57(3):711–7. 21907742 10.1016/j.appet.2011.08.017

[61] Gearhardt AN, Corbin WR, Brownell KD. Preliminary validation of the Yale Food Addiction Scale. Appetite. 2009;52(2):430–6. 19121351 10.1016/j.appet.2008.12.003

[62] Gearhardt AN, Corbin WR, Brownell KD. Development of the Yale Food Addiction Scale Version 20. Psychol Addict Behav. 2016;30(1):113–21. 26866783 10.1037/adb0000136

[63] Nair B. Clinical trial designs. Indian Dermatol Online J. 2019;10(2):193–201. 10.4103/idoj.IDOJ_475_18. 30984604 10.4103/idoj.IDOJ_475_18 PMC6434767

[64] Eccles M, et al. Research designs for studies evaluating the effectiveness of change and improvement strategies. BMJ Qual Saf. 2003;12(1):47–52. 10.1136/qhc.12.1.47 PMC1743658 12571345

